# Non-surgical body contouring and cellulite management: modalities, durability of results, and real-world longevity

**DOI:** 10.1016/j.jpra.2026.09.024

**Published:** 2026-09-23

**Authors:** Khalid Nabil Nagshabandi, Asem Shadid, Mohammed Al-Mashali

**Affiliations:** a Department of Dermatology, College of Medicine, King Saud University, Riyadh, Saudi Arabia; b Department of Dermatology, King Fahad Medical City, Riyadh, Saudi Arabia; c Department of Dermatology, College of Medicine, Al-Imam Muhammad Ibn Saud Islamic University, Riyadh, Saudi Arabia

**Keywords:** Non-surgical body contouring, Cellulite, Cryolipolysis, Subcision, Treatment durability

## Abstract

Demand for non-surgical body contouring and cellulite treatment continues to grow, yet the question patients ask first – how long will this last? – is poorly served by a literature dominated by 4- to 12-week endpoints. We reviewed clinical studies of device-based and injectable modalities reporting outcomes beyond the last treatment visit. Durability segregated by mechanism rather than by device category: interventions that irreversibly remove adipocytes or release fibrous septae sustained benefit for 12–36 months, whereas externally applied energy devices produced real but decaying change, typically attenuating within 3–6 months.

Non-surgical body contouring and cellulite devices have proliferated faster than the evidence needed to counsel patients about them. Most pivotal trials report endpoints at 4–12 weeks, and the field is conventionally organised by physics – radiofrequency (RF), high-intensity focused ultrasound (HIFU), high-intensity focused electromagnetic (HIFEM) stimulation, laser, acoustic – rather than by persistence of effect.^1^^,^^2^ Yet the question patients ask first is not how a device works but how long its result will last. We therefore interrogated the published literature along a single axis: documented durability.

We searched PubMed/MEDLINE, Embase and the Cochrane Library from inception to December 2025 for clinical studies of device-based or injectable body contouring and cellulite interventions in adults (≥10 participants) reporting objective or semi-quantitative outcomes beyond the last treatment visit. Twenty-six studies (14 body contouring, 12 cellulite) were synthesised narratively (Table 1).

**Table 1:** Durability of non-surgical body contouring and cellulite modalities, ordered by mechanism of action.

**Modality**	**Principal mechanism**	**Typical protocol**	**Longest well-documented follow-up**	**Durability verdict**
**Body contouring / localised adiposity**				
Cryolipolysis	Cold-induced adipocyte apoptosis, macrophage clearance	1–2 cycles of 35–120 min per site, single or repeated visits	3–4 months in most cohorts; isolated case follow-up at 6–9 years	Probably durable if weight stable; prospective long-term data lacking
HIFU	Focal thermal coagulative necrosis of fat	1–2 sessions to the waistline	24 weeks (sham-controlled)	Effect largely maintained to 6 months; beyond that unstudied
Radiofrequency (contact and non-contact)	Subdermal heating, collagen contraction, modest lipolysis	4–6 weekly sessions of ∼30 min	1–3 months	Short-term; maintenance anticipated
HIFEM ± synchronous RF	Supramaximal muscle contraction plus thermal fat effect	4–8 sessions over several weeks	∼3 months (MRI-documented)	Unknown beyond 3 months; no 12-month data
Low-level laser therapy	Transient adipocyte permeability, lipid mobilisation	6–16 sessions over 2–6 weeks	2–26 weeks	Partial rebound after treatment stops; adjunctive only
Injectable deoxycholic acid	Adipocytolysis with local fibrosis in treated plane	Up to 6 sessions ∼28 days apart (submental)	12 months after final session	Durable in responders; strongest long-term evidence here
**Cellulite**				
RF + infrared + mechanical manipulation	Dermal remodelling and mechanical tissue mobilisation	8–12 sessions over 4–6 weeks	∼3 months	Modest; maintenance required
Radial shockwave / acoustic wave therapy	Mechanical disruption of connective tissue	6–12 sessions over 6–7 weeks	3–6 months	Modest; maintenance required
Low-level laser therapy	Lipid mobilisation, dermal photobiomodulation	8–16 sessions over 4–8 weeks	∼8 weeks	Effect attenuates soon after treatment ends
Vacuum-assisted tissue-stabilised subcision	Controlled release of individual fibrous septae	Single in-office treatment	3 years	Durable; no further procedures needed in most subjects
1440-nm Nd:YAG side-firing laser septal release	Septal division plus targeted subdermal heating	Single treatment under local anaesthesia	12–24 months	Durable in appropriately selected patients
Needle-based acoustic subcision	Acoustic pulses disrupting septae without incision	Single in-office treatment	12 months	Durable to 1 year; multi-year data awaited
Collagenase clostridium histolyticum-aaes	Enzymatic cleavage of collagen-rich septae	Up to 3 sessions, 21 days apart	12 months	Durable to 1 year; bruising and injection-site pain expected

HIFU, high-intensity focused ultrasound; HIFEM, high-intensity focused electromagnetic; MRI, magnetic resonance imaging; Nd:YAG, neodymium-doped yttrium aluminium garnet; RF, radiofrequency.

Follow-up refers to the longest interval after the final treatment at which outcomes were formally assessed in the studies reviewed. Full study-level evidence tables are available from the corresponding author.

For adiposity, cryolipolysis has the largest treated population and the most consistent objective documentation, with ultrasound-measured fat-layer reductions of approximately 20–25% per treated site.^1^ Its mechanism – cold-induced adipocyte apoptosis followed by macrophage clearance – implies permanence provided weight remains stable, and isolated multi-year photographic follow-up supports this. Most published follow-up nevertheless stops at 3–4 months, so the durability claim currently rests more on biological plausibility than on prospective data. HIFU reduced waist circumference at 12 weeks with the effect largely maintained at 24 weeks.^1^ Non-contact RF and hybrid HIFEM plus synchronous RF platforms produce circumferential reduction and increased rectus abdominis thickness at 1–3 months, with essentially no published data beyond one year.^1^ Low-level laser therapy (LLLT) behaves as a mobilising adjunct rather than a definitive fat-removal tool: circumference falls during structured courses and partially rebounds once habitual diet and activity resume.^1^ The clearest longer-term signal in this group is injectable deoxycholic acid for submental fat, where a substantial proportion of phase 3 responders maintained improvement 12 months after the final session.^3^

The cellulite literature divides more sharply. Externally applied energy – RF with infrared light and mechanical manipulation, radial shockwave, acoustic wave therapy and LLLT – improves severity scores, skin elasticity and three-dimensional surface metrics, but characteristically over 3–6 months and with an explicit expectation of maintenance treatment.^2^ Interventions that mechanically or enzymatically interrupt the fibrous septae behave differently. Tissue-stabilised vacuum-assisted subcision maintained at least one grade of improvement in most women three years after a single in-office session, without additional procedures.^4^ Minimally invasive 1440-nm Nd:YAG side-firing laser septal release sustained improvement at 12–24 months, and needle-based acoustic subcision maintained response at 12 months.^2^ Collagenase clostridium histolyticum-aaes achieved responder rates superior to placebo with benefit generally maintained to 12 months, at the cost of predictable bruising and injection-site pain.^5^

The organising principle is therefore structural rather than technological. Treatments that irreversibly remove adipocytes or divide septae last; treatments that heat, cool superficially, mobilise or stimulate produce real but decaying change. Three consequences follow for practice. First, durability is a function of phenotype as much as device: the best sustained contouring outcomes occur in patients with focal adiposity, stable weight and contouring rather than weight-loss expectations, while in cellulite the dominant pattern – discrete tethered dimples versus diffuse laxity – should determine whether a septal-release or an energy-based approach is offered.^1^^,^^2^ Second, consent should be framed explicitly around durability versus invasiveness, since some patients will rationally prefer modest repeatable gains from external devices over bruising, downtime and local anaesthesia. Third, rare but high-impact events – paradoxical adipose hyperplasia after cryolipolysis, prolonged nodularity after septal-modifying procedures – belong in that conversation.^1^^,^^2^

These conclusions are constrained by the evidence available. This was a narrative rather than systematic synthesis; endpoints were heterogeneous, several cohorts were small and industry-sponsored, and participants were predominantly women with Fitzpatrick I–III skin and body mass index below 30, leaving durability in men, in higher BMI categories and in skin of colour largely uncharacterised. Standardised outcome sets combining circumference, imaging and patient-reported measures, prospective registries, and head-to-head comparisons of the durable options would materially change what can be promised at the bedside.

Until then, the honest answer to what actually lasts is mechanistic: destroy the adipocyte or release the septum and the result persists for years; anything else should be sold as maintenance.

## Ethical approval

Not required. This article is a synthesis of previously published literature and involved no human participants or identifiable patient data.

## Funding

This research did not receive any specific grant from funding agencies in the public, commercial, or not-for-profit sectors.

## Author contributions

KNN conceived the review, performed the literature search and synthesis, and drafted the manuscript. AS and MA contributed to study selection, data interpretation, and critical revision. All authors approved the final version for submission.

## Declaration of competing interest

The authors declare that they have no competing interests.

## Data Availability

All data discussed are derived from the published literature. The full study-level evidence tables prepared for the original submission are available from the corresponding author on request.
